# Na^+^/H^+^ exchanger NHE1 and NHE2 have opposite effects on migration velocity in rat gastric surface cells

**DOI:** 10.1002/jcp.25758

**Published:** 2017-02-21

**Authors:** Anja Paehler vor der Nolte, Giriprakash Chodisetti, Zhenglin Yuan, Florian Busch, Brigitte Riederer, Min Luo, Yan Yu, Manoj B. Menon, Andreas Schneider, Renata Stripecke, Katerina Nikolovska, Sunil Yeruva, Ursula Seidler

**Affiliations:** ^1^ Departments of Gastroenterology, Hemostatsis, Oncology and Stem Cell TransplantationMedical School of HannoverGermany; ^2^ Departments of Biochemistry, Hemostatsis, Oncology and Stem Cell TransplantationMedical School of HannoverGermany; ^3^ Departments of Hematology, Hemostatsis, Oncology and Stem Cell TransplantationMedical School of HannoverGermany

**Keywords:** cell migration, intracellular pH, mucosal protection, Slc9a1, Slc9a2, sodium hydrogen exchanger

## Abstract

Following superficial injury, neighbouring gastric epithelial cells close the wound by rapid cell migration, a process called epithelial restitution. Na^+^/H^+^ exchange (NHE) inhibitors interfere with restitution, but the role of the different NHE isoforms expressed in gastric pit cells has remained elusive. The role of the basolaterally expressed NHE1 (Slc9a1) and the presumably apically expressed NHE2 (Slc9a2) in epithelial restitution was investigated in the nontransformed rat gastric surface cell line RGM1. Migration velocity was assessed by loading the cells with the fluorescent dye DiR and following closure of an experimental wound over time. Since RGM1 cells expressed very low NHE2 mRNA and have low transport activity, NHE2 was introduced by lentiviral gene transfer. In medium with pH 7.4, RGM1 cells displayed slow wound healing even in the absence of growth factors and independently of NHE activity. Growth factors accelerated wound healing in a partly NHE1‐dependent fashion. Preincubation with acidic pH 7.1 stimulated restitution in a NHE1‐dependent fashion. When pH 7.1 was maintained during the restitution period, migratory speed was reduced to ∼10% of the speed at pH 7,4, and the residual restitution was further inhibited by NHE1 inhibition. Lentiviral NHE2 expression increased the steady‐state pH_i_ and reduced the restitution velocity after low pH preincubation, which was reversible by pharmacological NHE2 inhibition. The results demonstrate that in RGM1 cells, migratory velocity is increased by NHE1 activation, while NHE2 activity inhibit this process. A differential activation of NHE1 and NHE2 may therefore, play a role in the initiation and completion of the epithelial restitution process.

**Abbreviations**
BCECF‐AM: 2,7‐biscarboxyethyl‐5(6)‐carboxyfluorescein‐acetoxymethylester
BUME: bumetanide
CMV: cytomegalovirus
Cyt D: cytochalasin D
DIDS: 4,4′‐diisothiocyano‐2,2′‐stilbenedisulfonic acid
DiR: 1,1′‐dioctadecyltetramethyl indotricarbocyanine iodide = DilC_18_(7)
DMA: 5‐(N,N‐dimethyl)amiloride
DMEM: Dulbecco's Modified Eagle's Medium
DMSO: dimethyl sulfoxide
EGF: epidermal growth factor
EMT: epithelial‐mesenchymal transformation
FACS: fluorescence‐activated cell sorting
FCS: fetal calf serum
GFP: green fluorescent protein
HA: hemagglutinin
HEPES: 4‐(2‐hydroxyethyl)‐1‐piperazineethanesulfonic acid
HOE642: 4‐isopropyl‐3‐methylsulphonylbenzoyl‐guanidine methanesulphonate
IRES: internal ribosome entry site
MCT: monocarboxylate transporter
MitC: Mitomycin C
NBC: sodium bicarbonate cotransporter
NHE: Na^+^/H^+^ exchanger
NKCC: Na^+^K^+^2Cl^‐^ cotransporter
PBS: phosphate‐buffered saline
pH_i_: intracellular pH
qRT‐PCR: quantitative reverse transcription PCR
RGM1: rat gastric mucosal cell first
RPS9: ribosomal protein S9
TMA: tetramethylammonium
TRIS: tris(hydroxymethyl)aminomethane
VSVG: vesicular stomatitis virus G
WST‐1: water soluble tetrazolium
XbaI: Xanthomonas badrii restriction enzyme

## 1 INTRODUCTION

The gastrointestinal epithelium provides a physical barrier against noxious agents within the gastrointestinal lumen. During superficial wounding, rapid “healing” occurs in the healthy epithelium through the process of “gastrointestinal restitution,” where cells migrate from the depth of the gastric pits (Svanes, Ito, Takeuchi, & Silen, 1982, 1983), the basal part of the villi (Feil et al., 1989a) or colonic crypts (Feil et al., 1989b) to form a new, electrically tight epithelial layer within minutes of the initial injury (Svanes et al., 1982, 1983; Silen & Ito, 1985; Terano et al., 2001).

Early work established the importance of the luminal pH as well as high bicarbonate concentrations in the interstitium (for in vitro experiments) or in the systemic circulation (for in vivo experiments) in the process of epithelial restitution (Critchlow, Magee, Ito, Takeuchi, & Silen, 1985; Feil et al., 1989a; Svanes et al., 1983). Passive leakage (through the wounded surface) and active transepithelial transport of HCO_3_^−^ proved to be essential for rapid repair of superficial wounds of the gastric and duodenal mucosa in the presence of a fairly low luminal pH (Feil et al., 1989a; Guttu, Sørbye, Gislason, Svanes, & Grønbech, 1994; Svanes et al., 1983), while Na^+^/H^+^ exchange inhibition by amiloride reduced guinea‐pig gastric epithelial restitution (Joutsi, Paimela, Bhowmik, Kiviluoto, & Kivilaakso, 1996).

The gastric epithelium expresses high levels of NHE1, NHE2, and NHE4 (Rossmann et al., 2001). Cell fractionation studies (Rossmann et al., 2001) and immunohistochemical evidence (Stuart‐Tilley et al., 1994) suggest that, in addition to a particularly high basolateral expression of NHE1, gastric surface, and pit epithelial cells also display a high (Rossmann et al., 2001) apically localized (Xue, Aihara, Wang, & Montrose, 2011) expression of NHE2, while NHE4 is expressed strongly in parietal cells (Rossmann et al., 2001). NHE1 is crucial to accelerate migratory velocity and to maintain directed migration in fibroblasts, immune cells, and tumour cells, but this is in part due to NHE1‐dependent protein–protein interactions rather than NHE1‐mediated ion transport (Denker & Barber, 2002; Stock et al., 2008, 2005). Its role in gastric epithelial restitution is controversial (Hagen, Morrison, Law, & Yang, 2004; Ragasa et al., 2007; Yanaka et al., 2002). NHE2 is also highly expressed in the gastrointestinal epithelium, but 20 years after its molecular identification its physiological role is still not well understood (Rossmann et al., 2001; Seidler et al., 2011; Xue et al., 2011). Genetic deletion of NHE2 results in a severe gastric phenotype with a progressive loss of parietal and chief cells and the development of gastritis (Schultheis et al., 1998). The apical location of NHE2 also suggests it to be an unsuitable pH_i_‐regulatory mechanism in gastric surface cells, which face an acidic luminal milieu at their apical membranes. Xue et al. (2011) suggested that NHE2 may play a role during gastric wound healing, although the molecular mechanisms are not understood.

We therefore, searched for a suitable cell model to study the role of NHE2 in gastric epithelial restitution. Matsui and Ohno established the RGM1 cell line (**r**at **g**astric **m**ucosal cell first) from the glandular stomach of Wistar rats (Kobayashi et al., 1996). RGM1 cells are quickly‐growing nontransformed epithelial cells that stop proliferation when the monolayer is confluent. They have been extensively used as an in vitro model of gastric mucosa for the investigation of cytoprotective or harmful substances (Furukawa, Matsui, Suzuki, & Okabe, 1999; Nagano et al., 2012), proliferation or apoptosis (Kusuhara et al., 1998), inflammation (Tominaga et al., 2000), and gastric epithelial restitution (Furukawa et al., 1999; Hagen et al., 2004; Ragasa et al., 2007). After we had obtained the RGM1 cells from the RIKEN institute, we studied NHE isoform expression and found that RGM1 cells express NHE1 strongly, while minimally expressing other NHE isoforms. We therefore, studied the impact of NHE1 activity on migration by selective inhibition of the endogenous NHE1. The impact of NHE2 on RGM1 cell migration was analyzed by acute lentiviral NHE2 overexpression before the wounding assay. Fluorometric pH_i_‐metry in BCECF‐loaded RGM1 cells was utilized to verify NHE2 function in the transduced cells. A wound healing assay was established using fluorometric detection of wound closure in DiR‐loaded RGM1 monolayers (Menon, Ronkina, Schwermann, Kotlyarov, & Gaestel, 2009), which allowed an investigator‐independent assessment of restitution velocity and simultaneous measurement of controls.

## 2 MATERIALS AND METHODS

### 2.1 Reagents

Reagents for cell culture were purchased from PAA laboratories GmbH (Cölbe, Germany) unless otherwise stated. Sterile phosphate‐buffered saline (PBS) was obtained from Life Technologies GmbH (Darmstadt, Germany). DMEM (high glucose) and DMEM/Ham's F12 (1:1) and fetal calf serum (FCS) were bought from Biochrom AG (Berlin, Germany). Reagents for agarose gels, primers, and chemicals for fluorometry experiments were purchased from Sigma‐Aldrich Chemie GmbH (Munich, Germany). 2,7‐biscarboxyethyl‐5(6)‐carboxyfluorescein‐acetoxymethylester (BCECF‐AM), Cytochalasin D, and Mitomycin C were obtained from Applichem GmbH (Darmstadt, Germany). RNA isolation and qRT‐PCR kits were purchased from Qiagen GmbH (Hilden, Germany) and Eurogentec GmbH (Cologne, Germany). WST‐1 for cell viability assays was bought from F. Hoffmann‐La Roche AG (Mannheim, Germany). 1,1′‐dioctadecyltetramethyl indotricarbocyanine iodide (DiR) was kindly provided by Professor Matthias Gaestel (Hannover Medical School, Germany). HOE642 (cariporide) was a kind gift by Sanofi‐Aventis GmbH (Frankfurt, Germany). 5‐(N,N‐dimethyl)‐amiloride hydrochloride (DMA), G418 and epidermal growth factor (EGF) were purchased from Sigma‐Aldrich Chemie GmbH (Munich, Germany). Rat NHE1‐expressing PS120 (PS120/rNHE1) fibroblasts were a kind gift of Prof. Mark Musch, University of Chicago.

### 2.2 Cell culture

Rat gastric mucosal cells (RGM1) were grown on plastic in DMEM + Ham's F12 medium (1:1), supplemented with 1% penicillin–streptomycin and 20% FCS. PS120/rNHE1 cells were grown on plastic in DMEM (high glucose) supplemented with 10% FCS and containing 2000 μg/ml G418 for selection pressure. In a 5% CO_2_ atmosphere, these media displayed pH 7.4. Cells were grown on plastic or glass (for pH_i_ measurements), or on collagen for selected experiments, but seeding on collagen did not alter migration speed or NHE‐dependent regulation. An acidified medium (pH 7.1) for 24 hr preincubation was required for some of the experiments. Therefore, the complete cell culture medium (20% FCS) was adjusted to pH 7.1 by repeated titrations with 1 N HCl (5% CO_2_ atmosphere). Cells were passaged at 70–80% confluence and used for 26 passages. Cells were kept at 37°C with 5% CO_2_ and medium was replaced every 2–3 days.

### 2.3 RNA‐isolation, reverse transcription, and qRT‐PCR

RNA was isolated using the RNeasy® Mini Kit (Qiagen GmbH). RNA quality was ensured by capillary gel electrophoresis (QIAxcel® system, Qiagen GmbH). 1 μg RNA was reverse transcribed with the QuantiTect® Reverse Transcription Kit (Qiagen GmbH) according to the instruction manual. cDNA was diluted 1:10 and 5 μl of the dilution were used as a template for PCR. Each reaction contained 12.5 μl 2x MESA Green qPCR™ MasterMix (Eurogentec GmbH), an appropriate amount of primers (suppl. Table  1) and RNAse and DNAse free water up to a final volume of 25 μl. Identity and homogeneity of qRT‐PCR products were verified using DNA melting curves and 2% agarose gels. Data were analyzed using the efficiency corrected ΔΔCq method published by Pfaffl (Pfaffl, 2001). Primer efficiency was acquired from three different samples in separate qRT‐PCR runs using different serial dilutions. Efficiencies for different primers ranged from 1.92 to 2.04 (suppl. Table  1). The expression levels were calculated relative to the reference gene index (geometric mean of efficiency corrected Cq values of RPS9 and β‐actin).

### 2.4 WST‐1 cell viability assay

The reagent WST‐1 was used to determine cell viability according to the manufacturer's instructions. 4 × 10^4^ cells/well RGM1 cells were grown to confluence on a 96 well plate and incubated for 6–9 hr with the respective inhibitors. Afterwards, 10 μl WST‐1 was added to each well and 2 hr later absorbance was measured at 450 and 630 nm using the BioTek® Epoch Reader.

### 2.5 Cloning of the rat NHE2 full length gene into a lentiviral expression vector

Primers were designed based on the full length rat NHE2 (*Rattus norvegicus* solute carrier family 9 member 2) cDNA sequence which was obtained from the NCBI database. Monomeric HA was tagged at the 5′ end of the gene. XbaI restriction sites were added at the 5′ ends of both forward and reverse primers (non‐directional cloning). The sequence of the forward primer was: 5′ gac gca tct aga atg tac cca tac gac gtc cca gac tac gct ggc ccc tca ggc act gcg cac 3′, and the sequence of the reverse primer was: 5′ gac gca tct aga ggt tca tgg ctt ttc gtt gcc aag gcg gcc t 3′. These primers were successfully used to amplify the full length rat NHE2 gene with N‐terminal HA epitope tag (HA‐rNHE2) from cDNA isolated from RGM1 cells. The PCR product was digested with XbaI and ligated into the 11RRL‐CMV‐MCS‐IRES‐GFP lentiviral vector, which was also linearized with XbaI. The orientation of the gene was confirmed by restriction digestion and by sequencing. HA‐tagged NHE2 protein expression was analyzed by Western blot using anti‐HA antibody in HEK 293T cells, and the HA‐NHE2 band at ∼90 kDa coincided with the theoretical molecular weight of 91.41 kDa (suppl. Fig.  1).

### 2.6 Recombinant, lentivirus‐mediated NHE2 overexpression in RGM1 cells

Stripecke recently described the lentivirus production (Stripecke, 2009). HEK 293T cells were transduced with the lentiviral vector containing HA‐rNHE2 and the control backbone plasmid along with packaging plasmids. 36 hr after transduction, supernatants were collected and centrifuged at 25,000 rpm for 3 hr to concentrate the virus. The pellet was re‐suspended in 1 ml PBS and stored at −80°C. HA‐rNHE2 lentivirus and control virus were added to confluent monolayers. Transductions were performed for 24 hr in 20% FCS medium supplemented with 5 μg/ml polybrene to improve efficiency. Prior to subsequent experiments, RGM1 cells were washed once with PBS. Transducing 1 × 10^5^ RGM1 cells with 20 μl concentrated HA‐rNHE2 containing lentivirus provided high GFP expression (suppl. Fig. 2A and B) as well as high NHE2 activity (Fig. 7). Flow cytometry detected GFP staining in ∼70–80% of HA‐rNHE2 overexpressing cells and in >90% of cells transduced with the control vector (suppl. Fig. 2).

### 2.7 Na^+^‐dependent pH recovery and steady‐state pH_i_

RGM1 cells and PS120/NHE1 cells were plated onto 25 mm glass coverslips. For the assessment of the inhibitory effect of HOE642 on RMG1 and PS120/NHE1 NHE activity, the cells were measured at 90% confluency, without serum starvation. For all other experiments, the cells were studied at identical conditions at which the wounding was performed (after reaching confluence and 24 hr serum starvation). The cells were incubated with 5 μM BCECF‐AM in 1× NaCl solution for 30 min at room temperature. Coverslips were placed in a temperature‐controlled perfusion chamber. Both for nontransfected and GFP‐transfected cells, background fluorescence was recorded prior to BCECF loading, in nontransfected cells in aliquots what were not used for BCECF measurements, and in GFP‐transfected cells in the same sample that was used after BCECF‐loading in the chamber. GFP‐related fluorescence signal was low (<20‐fold) compared to the BCECF‐related signal. BCECF was excited alternately at 440 and 495 nm wavelength and the intensity of emission signals was recorded at 530 nm. See supplementary Table 2 for details on composition of solutions used during the following experimental setups: i) the intracellular steady‐state pH was measured by perfusing the cells with 1x NaCl solution (gassed with CO_2_) for at least 20 min until the emission signals was stabilized, then calibration was performed with 10 mM Nigericin/high K^+^ and different pH solutions close to the anticipated steady‐state pH; ii) the initial Na^+^‐dependent pH recovery from an NH_4_Cl‐induced acid load was measured in the absence or presence of CO_2_/HCO_3_^−^ (columns 1× NaCl O_2_ or CO_2_), and is given as ΔpH/min, as previously described (Xia et al., 2014).

### 2.8 Fluorescence‐based wound healing assay

Wound healing assays were performed as previously described (Menon et al., 2009), with slight modifications. Briefly, 4 × 10^6^ RGM1 cells in 3 ml cell culture medium were stained with 4.5 μl infra‐red fluorescent dye DiR for 15 min at 37°C. After washing with PBS, cells were re‐suspended in 10 ml cell culture medium. Cells were seeded with a density of 4 × 10^4^ cells in 96 well plate (100 μl/well). With this seeding density, cells formed a confluent monolayer with homogenous staining. To arrest proliferation (if desired), cells were deprived of serum (0% FCS), which did not induce apoptosis during the time of the experiment (suppl. Fig. 3). 24 hr prior to wounding RGM1 monolayers were scratched using a 100 μl pipette tip to create the “wound.” Culture medium was replaced with fresh medium containing appropriate amounts of inducers or inhibitors. Cells were incubated for one hour to recover. Afterwards, initial scans and further scans after 6 hr (during this time migration velocity is linear over time) were taken by infra‐red fluorescence scanner (Odyssey®, Li‐Cor Biosciences) at 800 nm emission wavelength. The fluorescence scans were converted into 8‐bit data files using ImageJ (suppl. Fig. 4). Then, a threshold was adjusted, changing all grey values below the threshold to black and all above the threshold to white. On this basis a black and white image was created and the area covered by black pixels, representing the scratch, could be measured automatically by ImageJ. Individual threshold correction was performed as described in suppl. Figure 5. Relative wound healing velocity was calculated by determining the migration index as described (Menon et al., 2009) and presenting the data as percentage in relation to the individual control converted to 100%, to account for possible inter‐assay variation. The assay was compared with the classical well established wound closure assay based on time‐lapse video microscopy. Epidermal growth factor (EGF) and fetal calf serum (FCS) dose‐dependently stimulated wound closure in RGM1 cells (suppl. Fig. 6A), similar to recently published data (Nakamura, Takahashi, Matsui, & Okabe, 1998). Cytochalasin D, an inhibitor of actin assembly and inducer of actin depolymerisation, strongly reduced migration (suppl. Fig. 6B).

### 2.9 Determining restitution velocity by time lapse microscopy

Time‐lapse video migrations were performed as follows: 2.5 × 10^5^ RGM1 cells were plated per 25 ml flask and grown for 2 days until reaching confluence. The confluent monolayers were scratched with a 100 μl pipette tip and mounted in a heated chamber on an inverted microscope. Images were taken every 10 min for 3–6 hr and time lapse videos were generated using the ImageJ program. To determine wound healing velocity, the area of the scratch was measured with the ImageJ program. The decrease of the area over time allowed for calculation of wound healing velocity.

### 2.10 Statistics

Data are expressed as mean with SEM, if not otherwise indicated. Experiments were usually performed three times (*n* = 3, and within one experiment, several different wells (migration), or coverslips (pH_i_‐measurements) were assessed. For the pH_i_ measurements by fluoromicroscopy, 2–3 coverslips were analyzed per condition and cell passage, and *n* is the number of individual passages investigated. Wound healing assays were carried out in two to three different passages with five to ten wells for each repetition (*n* = 15–30). Data were compared by two‐tailed t‐test (two groups) or one‐way ANOVA (more than two groups) with subsequent Dunnett's test or Bonferroni's multiple comparison test. *p < 0.05, **p < 0.01, ***p < 0.001.

## 3 RESULTS

### 3.1 RGM1 cells express predominantly NHE1 mRNA and transport activity

**Figure 1:**
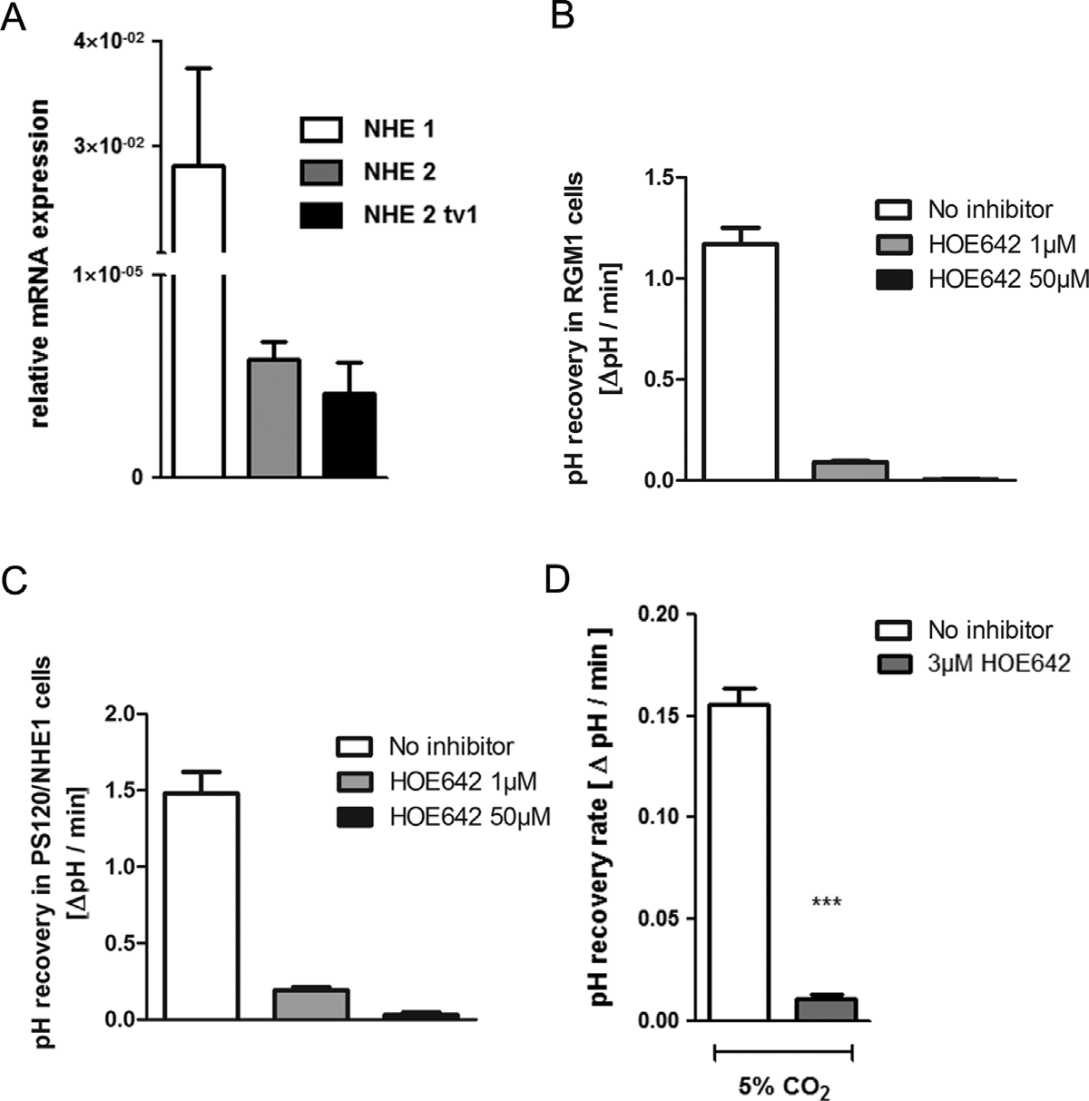
RGM1 cells express predominantly NHE1 mRNA and exchange activity (A) Expression of NHE1 mRNA and both rat NHE2 transcript variants (NHE2) as well as only the longer variant of NHE2 (NHE2 tv1) was determined in RGM1 cells grown in 20% FCS in relation to marker genes as indicated in the methods. (B) Bars show the Na+‐dependent pHi recovery as △pH/min in RGM1 cells during recovery from ammonium prepulse induced intracellular acidification in the absence of inhibitors, and in the presence of 1 and 50 μM HOE642. (C) The same experimental protocol was performed in PS120/rNHE1 fibroblast, which only express functional NHE1, demonstrating that 1 μM HOE642 does not fully inhibit NHE1. (D) For these experiments, the RGM1 cells had been studied under the same conditions in which the wound healing was performed (24 hr serum‐starvation, CO_2_/HCO_3_^−^containing perfusate), and the cells were only acidified to pHi ∼6.5–6.7. However, the near‐total inhibition of pHi‐recovery by 3 μM HOE642 shows that NBCs play no role in RGM1 cell pHi recovery. *n *= 6–8,***p < 0.001

As shown in Figure 1A, The RGM1 cell line was found to express NHE1 mRNA at far higher levels than NHE2. Expression of NHE3 and NHE8 was also extremely low (near the detection limit), and NHE4 expression was not detected at all (data not shown). Incubation in 20% serum or in slightly acidic medium for 24 hr did not increase NHE1 and NHE2 mRNA expression significantly (suppl. Fig. 7). Given the fact that in PS120 fibroblasts, NHE2 has a far shorter membrane halflife than NHE1 (Cavet et al., 2001), it is not likely that these very low NHE2 mRNA expression level lead to any functional NHE2 activity in the membrane.

To test this hypothesis, the functional activity of the Na^+^/H^+^ exchangers was determined as initial rate of Na^+^‐dependent pH recovery from an acute intracellular acid load. Figure 1B shows the pH_i_‐recovery of RGM1 cells grown in FCS and at the day of confluency, from an NH_4_‐prepulse‐mediated acidification to ∼6.0, which maximally activates NHEs including NHE1, in the absence of inhibitors, as well as 1 and 50 μM HOE642, in an O_2_‐gassed Hepes/Tris buffered perfusate (see suppl. Table 2). 1 μM reduced pH_i_ recovery rate to 91 ± 8%, and 50 μM HOE642, which would also inhibit NHE2, if present (Scholz et al., 1995), in this concentration, to virtually zero.

In order to distinguish whether the very small difference in pH‐recovery rates between the presence of 1 and 50 μM HOE642 was due to an incomplete inhibition of NHE1 by 1 μM HOE642, or due to NHE activity of another isoform, we measured acid‐activated Na^+^‐dependent pH_i_‐recovery in PS120/rNHE1 fibroblasts under identical experimental conditions. This cell line only expresses functional NHE1 (Bookstein et al., 1996, 1997). As displayed in Figure 1C, 1 μM HOE642 inhibited 88 ± 12% of NHE1 activity under the chosen experimental conditions, while 50 μM was fully inhibitory.

We also studied pH‐recovery in the identical experimental conditions under which the wound healing experiments were performed (24 hr‐serum starved, in CO_2_/HCO_3_^−^) (Fig. 1D). The cells were acidified to pH_i_ ∼6.5, which does not maximally activate NHE1 but is within the linear fluorescence‐pH relationship for BCECF. For these experiments, we increased the HOE642 concentration for a slightly better NHE1 inhibition but no inhibition of NBCs. This inhibited pH_i_‐recovery rates to 90 ± 4% of the rates in the absence of inhibitors (Fig. 1D). Although RGM1 cells express some NBC1, as detected by PCR (Hayashi et al., 2008), NBCs do not substantially participated in pH_i_‐recovery under these experimental conditions.

### 3.2 NHE1 activity is not essential for restitution velocity in RGM1 cells at pH 7.4

**Figure 2:**
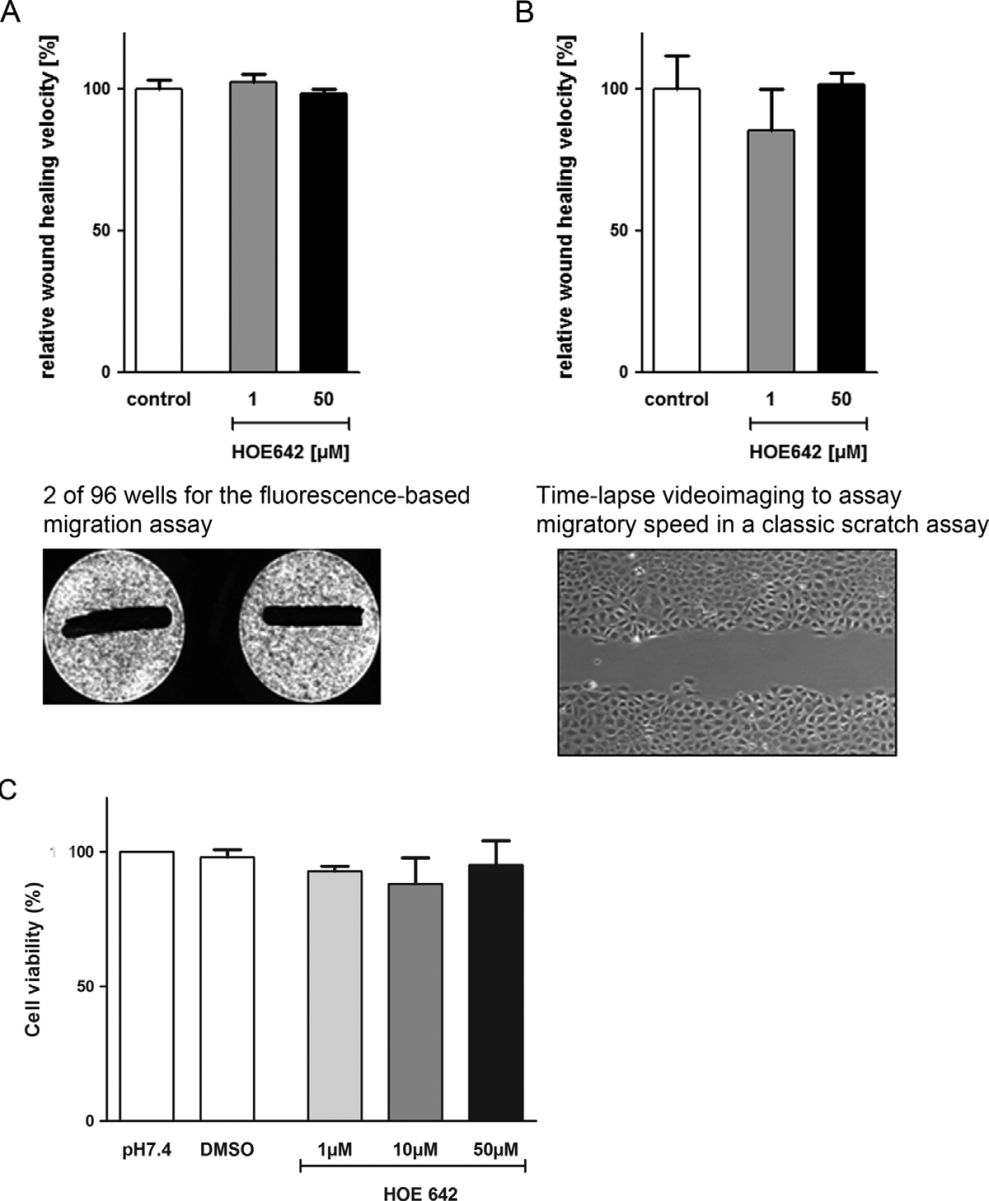
NHE1 inhibition does not affect RGM1 wound healing in the absence of FCS (A) Wound healing velocity was determined by the fluorescent DiR based wound healing assay as described in material and methods including suppl. Figures 4, 5, and 9. NHE1 was inhibited by up to 50 μM HOE642 to guarantee 100% NHE1 inhibition. RGM1 cells were deprived of serum during migration (0% FCS). Bars show the relative wound healing velocity in percent, compared to the respective vehicle control. The image below the bar graph displays two representative wells of a 96 well plate. The fluorescent monolayer is shown as white sheet, while the scratch demarcates itself in black. (B) The results were verified using the well established wound healing assay by time lapse video microscopy. The image displays a representative part of the scratch which was analyzed in the time lapse video assay as described (Schwab et al. 2005). HOE642 did not significantly change restitution velocity even at high concentrations, using either restitution assay. (C) WST cell viability assay shows that the incubation with HOE642 in the tested concentrations does not decrease cell viability during the time of the experiment

We next determined the involvement of NHE1 activity in RGM1 wound healing in the absence of growth factor stimulation. Serum starvation with 0% FCS for 24 hr eliminated proliferation (suppl. Fig. 7A) but did not induce apoptosis (suppl. Fig. 7B). Up to 50 μM HOE642 did not change restitution velocity during wound healing significantly (Fig. 2A). Collagen coating did not influence the results (suppl. Fig. 8C). Similar experiments were performed by quantifying migration by video microscopy. In accordance with data derived from the fluorescence‐based scratch assay, there was no significant effect of HOE642 on restitution (Fig. 2B). This indicates that serum‐starved RGM1 cells migrate at slow speed (even when NHE1 is completely quiescent).

**Figure 3:**
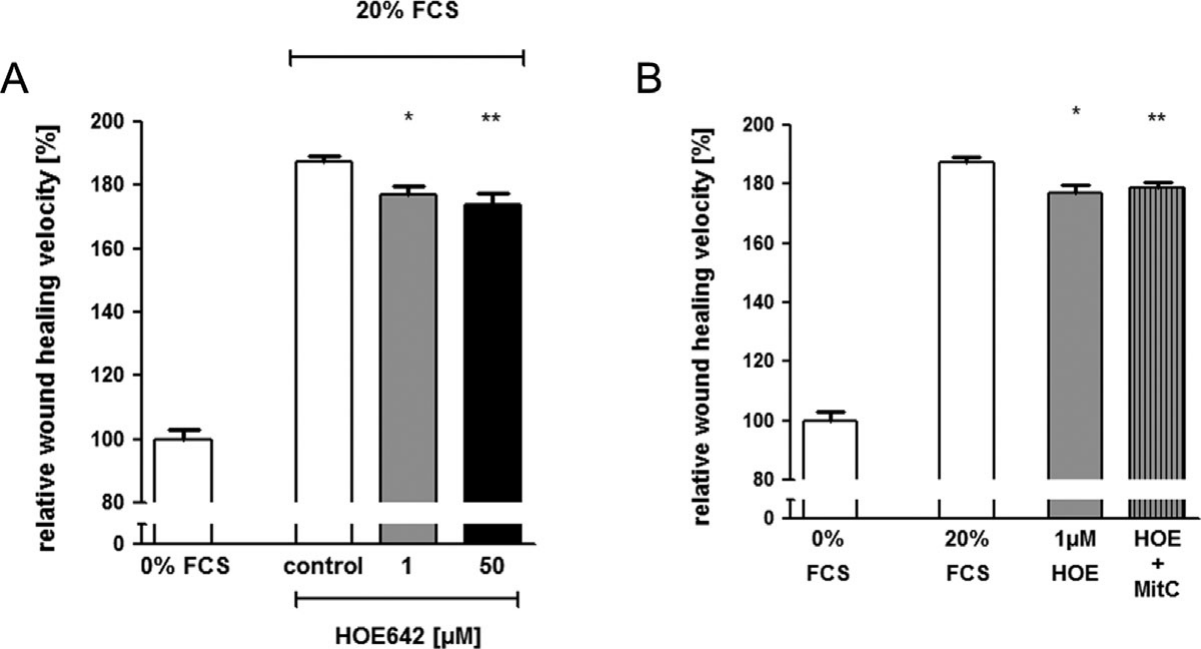
NHE1 activity accelerates migration velocity in the presence of FCS (A) Restitution velocity was stimulated by 20% FCS to almost 200% of the control (with 0% FCS). This increase was inhibited by about 10–14% when the intrinsic NHE1 was inhibited with HOE642 1–50 μM. (B) Application of 2 μM MitC (to inhibit proliferation) and 1 μM HOE642 did not reduce healing velocity further than HOE642 alone, demonstrating that the effect of FCS on migration velocity were not related to its effect on proliferation during the 6 hr measurement time. Statistical significance was determined by comparison to the corresponding controls using one way ANOVA with Dunnett‘s post test or two tailed t test, respectively. *p < 0.05, **p < 0.01

Next, restitution velocity was determined in RGM1 cells that were maintained in 20% FCS medium prior to and during the assay. The presence of 20% FCS stimulated restitution velocity twofold, and the inhibition of NHE1 by HOE642 decreased migratory speed by approx. 10% (Fig. 3A) [1 μM (inhibits NHE1 >80%) to 50 μM (inhibits NHE1 completely but would also affect NHE2 and presumably NHE8, if present) HOE642]. Inhibition of proliferation by 2 μg/ml of the DNA crosslinker Mitomycin C (MitC) (Nguyen et al., 2007) did not further reduce restitution velocity, suggesting that the stimulatory effect of FCS on wound healing was not by stimulation of proliferation (Fig. 3B).

### 3.3 Acid preincubation accelerates RGM1 restitution velocity in a NHE1‐dependent fashion

**Figure 4:**
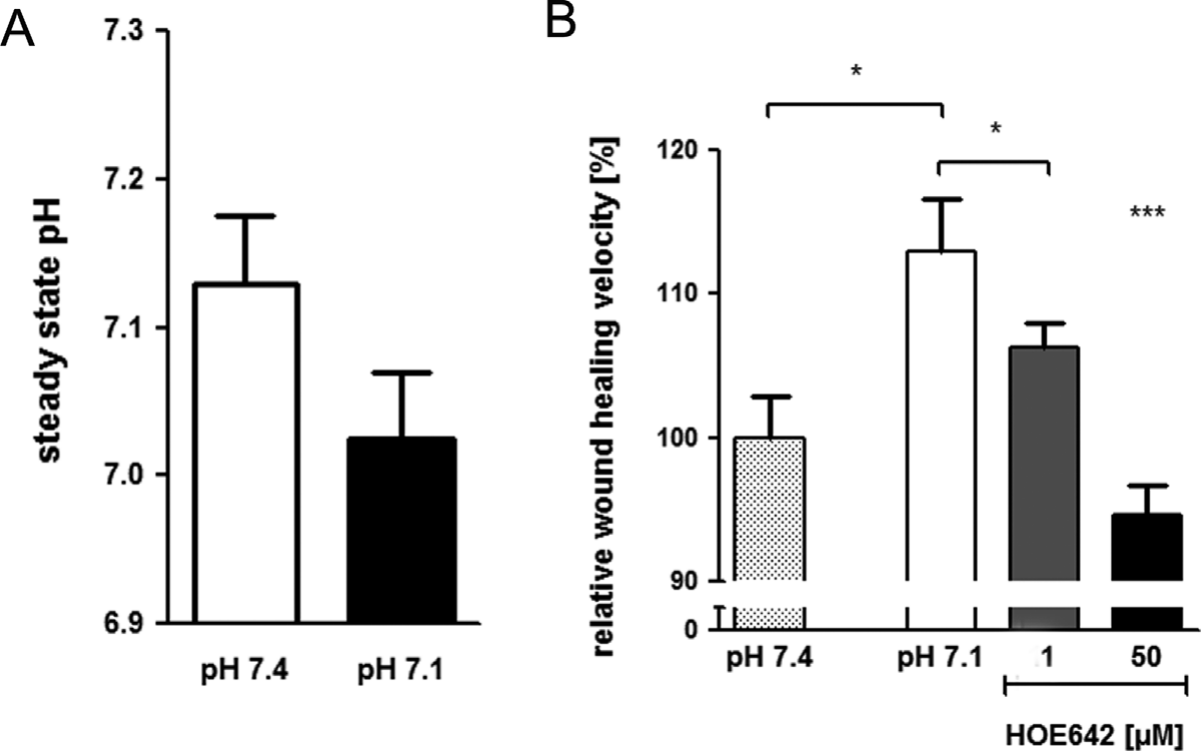
Acid preincubation induces RGM1 wound healing via stimulation of NHE1 24 hr prior to the scratch assay, RGM1 cells were incubated with acidic pH 7.1 to induce NHE activity. (A) Bars show the steady state pHi of RGM1 cells during incubation with physiological (pH 7.4) or acidic (pH 7.1) medium. Incubating RGM1 cells at pH 7.1 acidifies intracellular pH from about pH 7.13 to pH 7.03. *n* = 4. (B) Bars show the relative wound healing velocity in percent of the control with pH 7.4 preincubation (100%). pH 7.1 preincubation (but restitution in pH 7.4) increased migration velocity of RGM1 cells, and this increase was eliminated by complete NHE1 inhibition using 50 μM HOE642. The different efficacy of 1 and 50 μM HOE642 in inhibiting migration velocity is likely due to the fact that 1 μM HOE642, added as a stock after the medium change to pH 7.4, resulted in an incomplete NHE1 inhibition at the start of the wound healing process. Statistical significance was determined by one way ANOVA with Bonferroni's multiple comparison post test. *p < 0.05, ***p < 0.001

Possibly, the NHE1 is relatively quiescent in the migrating RGM1 cells at the extracellular pH of 7.4. Acid preincubation has been described to activate Na^+^/H^+^ exchange (Horie, Moe, Tejedor, & Alpern, 1990; Moe et al., 1991). RGM1 cells were therefore, incubated for 24 hr at mildly acidic pH 7.1 prior to wounding, and restitution was assayed at pH 7.4. Administration of pH 7.1 for 24 hr did not reduce cell viability, as confirmed by WST‐1 assays (Fig. 4A), but decreased the steady‐state pH_i_ from pH 7.13 ± 0.04 to 7.03 ± 0.05 (Fig. 4B) without significant changes in NHE1 and NHE2 mRNA levels (suppl. Fig. 6). Restitution velocity increased significantly when cells were preincubated with pH 7.1, and this was inhibited by 1 and 50 μM HOE642 (Fig. 4B). In this experimental series, the observed difference in inhibition of migratory velocity with 1 versus 50 μM HOE642 is possibly explained by a faster and more complete inhibition of NHE1 (because of the diffusion delay of the substance when pipetted to the medium to reach to the transporter) during the pH‐recovery period after the medium switch from pH 7.1 to pH 7.4, when 1 and 50 μM HOE642 was added as a small volume of a 100‐fold higher stock solution after the medium change.

### 3.4 Perpetuation of acidic pH throughout the assay strongly inhibits RGM1 wound healing

**Figure 5:**
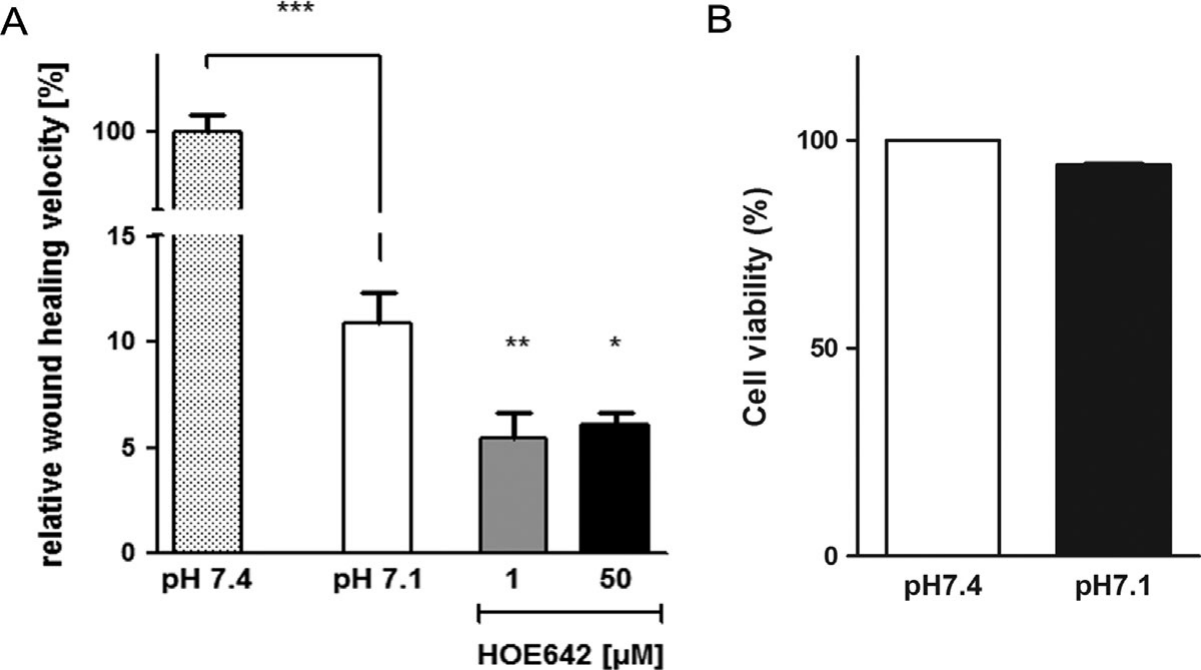
Migration velocity is strongly inhibited at medium pH of 7.1 (A) When the RGM1 cells were cultured at pH 7.1 throughout the scratch assay (pH 7.1), wound healing velocity was inhibited by almost 90% compared to the velocity of the control at pH 7.4. Adding 1–50 μM HOE642 further significantly reduced the residual restitution velocity. Bars show the relative wound healing velocity in percent compared to the vehicle control with pH 7.4 (100%). (B) Cellular viability remained unaffected by incubation at a medium pH of 7.1, *p < 0.05, **p < 0.01, ***p < 0.001

To investigate the effect of mildly acidic pH on restitution, pH 7.1 medium was also maintained throughout the whole wound healing procedure. The perpetuation of acidic pH strongly inhibited RGM1 velocity by almost 90%. Inhibition of NHE1 (1–50 μM HOE642) or addition of 100 μM DMA further reduced the restitution velocity significantly (Fig. 5).

### 3.5 Lentiviral NHE2 expression increases both pH_i_ recovery rates as well as steady state pH_i_ before and after incubation in mildly acidic pH

**Figure 6:**
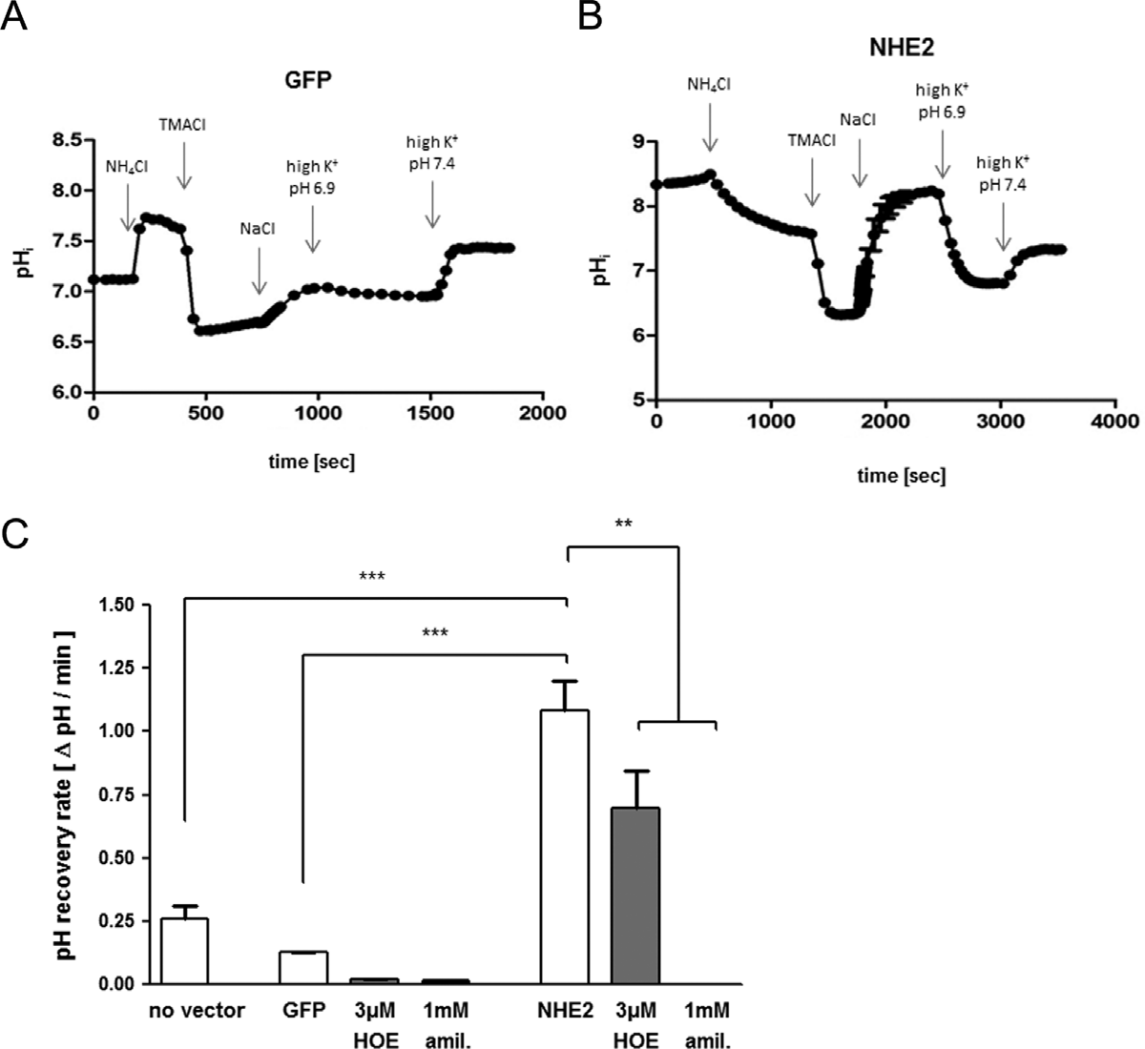
Lentiviral expression of rat NHE2 in RGM1 cells resulted in enhanced pHi recovery with a NHE2 typical inhibitor profile. (A and B) Two exemplary pHi recovery curves of empty vector/GFP controls (A) and of NHE2 overexpressing RGM1 cells (B). The arrows mark changes of perfusion solutions (exact composition in suppl. Table 2). NHE2 overexpressing cells display a higher “starting“ pHi as well as a higher recovery rate after intracellular acidification. (C) pHi recovery in the first minute after sodium addition. “No vector” cells were not transduced at all. In control vector transduced cells (GFP), 3 μM HOE642 as well as 1 mM amiloride both virtually eliminated pHi recovery. NHE2 overexpressing cells (NHE2) display a much higher pHi recovery, and only approx. 1/3 of this rate is inhibited by 3 μM HOE642. Full inhibition was achieved by 1 mM amiloride. Statistical significance was determined by one way ANOVA with Bonferroni's multiple comparison post test. *n *= 6, **p < 0.01, ***p < 0.001

In contrast to RGM1 cells, native gastric surface cells have high NHE2 mRNA expression levels (Rossmann et al., 2001) and we were interested in the role of this NHE2 in the gastric restitution. NHE2 was therefore, expressed in RGM1 cells via lentiviral gene transfer. In order to evaluate the presence of membrane‐resident NHE2 expression, NHE2‐mediated pH_i_‐recovery was assessed in BCECF‐loaded GFP/NHE2‐ or GFP/empty vector transduced cells using the ammonium prepulse technique. 3 µM HOE642 was applied to inhibit most NHE1 activity with minimal effect on the NHE2 activity, while 1 mM amiloride was applied to inhibit all NHE activities including that transferred by the heterologously expressed NHE2. Figure 6A and B shows representative pH_i_‐traces of an ammonium prepulse experiment in GFP/empty vector transduced and GFP/NHE2‐transduced cells in the absence of inhibitors. NHE2‐transduced cells display a markedly higher initial pH and a faster pH_i_‐recovery. The GFP/empty vector transduced cells displayed a somewhat slower pH_i_‐recovery than nontransduced RGM1 cells, but as in the nontransduced cells (Fig. 1D), pH_i_‐recovery was almost fully inhibited by NHE1 specific inhibition by 3 μM HOE642, which had the same effect as 1 mM amiloride (blocks all NHEs) in these cells (Fig. 6C). In contrast, 3 μM HOE642 resulted in a partial and 1 mM amiloride in a complete inhibition of pH_i_ recovery in NHE2‐transduced RGM1 cells. The percentage of the pH_i_‐recovery rate that was sensitive to 3 μM HOE642 was higher in NHE2‐transduced than GFP‐transduced cells, indicating the fact that even 3 μM HOE642, along with almost complete NHE1 inhibition, already inhibits a fraction of NHE2 activity (Scholz et al., 1995). These experiments indicated robust acid activated NHE2 activity in a sufficiently high number of cells to significantly alter the pH_i_ regulatory properties of the monolayers.

**Figure 7:**
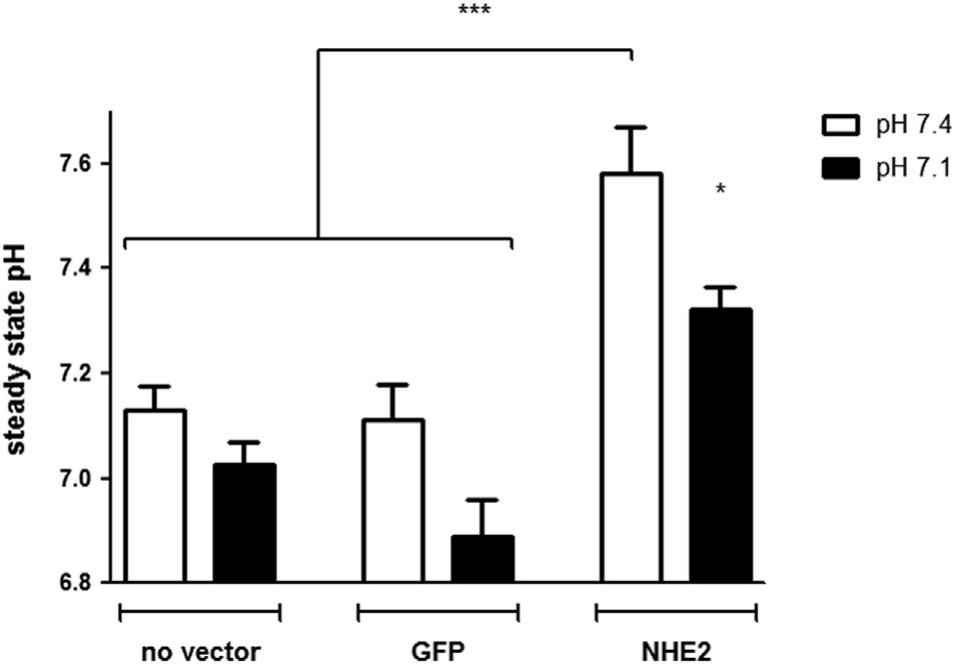
NHE2 overexpression results in a decrease of migration velocity Wound healing assays were done in NHE2 overexpressing RGM1 cells (A) in the absence of growth factors (0% FCS) and (B) in 0% FCS, after acid preincubation (pH 7.1). Bars show the relative migration velocity of RGM1 cells transduced with the GFP control vector (GFP) or with the NHE2 containing vector (NHE2) compared to non transduced RGM1 cells (no vector). (A) With 0% FCS neither intrinsic NHE1 nor overexpressed NHE2 displayed an influence on migration velocity. (B) After acid preincubation, NHE2 overexpression reduced migration by 52%. This decrease was unaltered by NHE1 inhibition (3 μM HOE642) but was eliminated by additional NHE2 inhibition(50 μM HOE642). ***p < 0.00

### 3.6 NHE2 overexpression does not influence basal restitution but strongly inhibits acid‐induced migratory velocity

**Figure 8:**
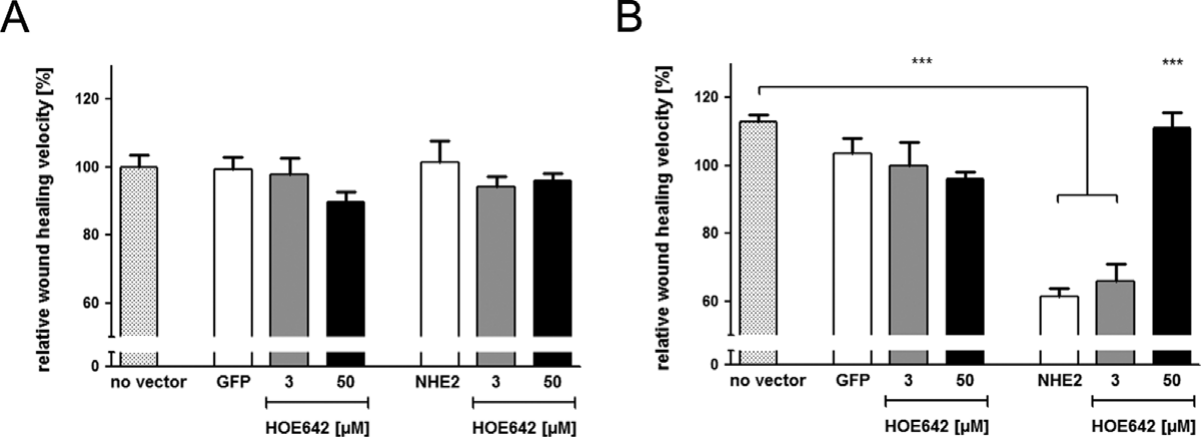
Steady state pHi after 24 hr incubation in pH 7.4 and pH 7.1 in control, in GFP and in NHE2/GFP transduced RGM1 cells. Cells were incubated for 24 hr in complete cell culture medium (20% FCS) at pH 7.4 (white bars) or pH 7.1 (black bars). Subsequently, they were loaded with BCECF and the steady‐state pHi was assessed in CO_2_‐equilibrated medium with pH 7.4 and 7.1. Non transduced RGM1 cells (no vector) were compared to those transduced with the GFP containing control vector (GFP) and those transiently overexpressing NHE2 (NHE2). NHE2 overexpression resulted in a significantly higher steady state pH both in pH 7.4 and 7.1 medium. *p < 0.05, ***p < 0.001

We decided to test the effect of NHE2 activity in the “basal” setting and after “preactivation” by 24 hr incubation at pH 7.1. Before doing so, we firstly assessed the steady‐state pH_i_ in control, GFP/empty vector transduced cells, and GFP/NHE2‐transduced cells (Fig. 8). Steady‐state pH_i_ was significantly lower after 24 hr preincubation in medium with pH 7.1 compared to pH 7.4, both in nontransduced, in GFP/empty vector and GFP/NHE2‐transduced cells. Despite the markedly higher steady‐state pH_i_ in NHE2‐expressing RGM1 cells, the restitution velocity was not affected by either empty vector or NHE2 transduction in 0% FCS (Fig. 8A). However, when cells were subjected to 24 hr preincubation at pH 7.1 medium, the subsequent restitution velocity was significantly decreased in GFP/NHE2‐transduced cells, while it was not influenced by GFP/empty vector transduction. This reduction was unaltered by NHE1 inhibition with 3 μM HOE642, but was eliminated by blocking NHE2 activity with 50 μM HOE642 during the migration assay (Fig. 8B), suggesting that NHE2 activity significantly decreased migration velocity.

## 4 DISCUSSION

The nontransformed gastric epithelial cell line RGM1 cells expressed high NHE1 mRNA levels, but extremely low NHE2, NHE3, NHE8 levels and no NHE4 mRNA. pH_i_‐recovery from an acid load was performed almost exclusively by NHE1, with no evidence for NBC‐ or by other NHE isoforms‐mediated pH_i_‐recovery (Fig. 1). RGM1 cells therefore, represented an good model to study the effects of endogenous NHE1 in gastric epithelial “sheet migration,” a process in which the cells maintain their cell‐cell contacts during migration, but for the study of NHE2, the RGM1 cell had to be transfected.

RGM1 cells were able to migrate even in the absence of growth factors and serum. This relatively slow migratory rate was not affected by complete NHE1 inhibition (Fig. 2). In the presence of serum, restitution velocity was almost twice as high as that of serum starved cells, and NHE1 inhibition reduced this migration speed by 10–15%. The experiments demonstrate that high concentrations of the various growth factors present in fetal calf serum stimulate high migration velocity in RGM1 cells despite a quiescent NHE1. Thus, although growth factors stimulate NHE1 (Sardet, Counillon, Franchi, & Pouysségur, 1990) the major part of the stimulatory effect of serum on RGM1 migration velocity is not via stimulation of NHE1.

Twenty‐four hour mild “acid stress” (medium pH 7.1) has been shown to stimulate NHE1 without reducing cell viability (Horie et al., 1990; Moe et al., 1991, Fig. 4A). Twenty‐four hour preincubation at medium pH 7.1 with subsequent migration at medium pH 7.4 stimulated restitution velocity in nonproliferating RGM1 cells (0% FCS) by approx. 15%, and this increase was fully inhibited by NHE1 inhibition. Possibly NHE1 is quiescent in nonproliferating RGM1 cells, and prestimulation of NHE1 activity prior to wounding (by acid or by FCS) may then result in a higher restitution velocity. An extracellular pH of 7.1 maintained during the restitution phase, on the other hand, inhibited wound closure almost completely, and the residual slow migratory speed was strongly dependent on NHE1 activity (Fig. 5). The finding that NHE1 activity becomes more important for restitution in an acidic environment is in line with previous observations of the sensitivity of gastric restitution to the luminal pH (Critchlow et al., 1985; Guttu et al., 1994; Silen & Ito, 1985). Because of the remarkable ability of the gastrointestinal epithelium to create an alkaline micromilieu above the epithelium (reviewed in: Seidler et al., 2011), to maintain this alkaline micromilieu (Wallace & McKnight, 1990) or even to increase its alkalinity in the event of wounding (Demitrack, Soleimani, & Montrose, 2010), it has previously escaped notice just how extremely sensitive the migratory process of gastric surface epithelial cells may be to even such a slight decrease in extracellular pH to 7.1. Melanoma cells, for example, have their optimal migratory speed at a pH ∼7 (Stock et al., 2005), and prostate cancer cells at pH 6.6 (Riemann et al., 2016).

Murine, rat, and rabbit gastric surface cells all display high endogenous NHE1 as well as NHE2 expression (Rossmann et al., 2001; Stuart‐Tilley et al., 1994). Since RGM1 cells turned out to have almost 1,000 fold lower NHE2 than NHE1 mRNA expression levels, we expressed NHE2 using a lentiviral bicistronic vector for NHE2 and GFP individually, resulting in high GFP/NHE2 expression levels that were rapidly lost during subsequent passages. The lentiviral NHE2 transduction without subsequent antibiotic selection was chosen in order not to end up with cell clones of different cellular size and proliferating capacity as compared to the mother cell line, as can be the case when stable cell lines are created that lack or overexpress NHEs (Schwab et al., 2005). Transduction efficiency was 70–80%, which resulted in a robust increase in the steady‐state pH_i_, as well as high NHE2‐mediated pH_i_‐recovery rates in the transduced RGM1 cells.

NHE2 overexpression resulted in a significantly higher pH_i_ than that displayed by the nontransduced or empty‐vector transduced cells (Fig. 7), yet no significant difference in migratory speed was observed at medium pH of 7.4 (Fig. 8A). Unexpectedly, NHE2‐transduced cells had a markedly slower restitution velocity than empty vector or nontransduced cells after acid preincubation. One possible explanation might be that they did not get acid‐activated due to their high pH_i._ However, when we measured the steady‐state pH_i_ after the 24 hr pH 7.1 incubation, we found that both empty vector‐ and nontransduced cells, as well as NHE2‐transduced cells had obtained a lower steady‐state pH_i_ (Fig. 7). The addition of a NHE2‐inhibiting concentration of HOE642 only during the restitution period, not during the acid preincubation period, completely abolished the reduction of restitution velocity, while a NHE1‐inhibiting concentration did not. In contrast, HOE642 displayed the same slightly inhibitory effect in the empty vector controls as was observed previously in the acid‐preincubated RGM1 cells (Fig. 8B, Fig. 5). This suggests that NHE2 function indeed inhibits restitution velocity in acid‐preincubated RGM1 cells.

What could be the mechanism of an inhibition of restitution velocity in gastric epithelial cells by NHE2 activity? Current evidence suggests that NHE1 and NHE2 are located in different membrane areas in gastric epithelial cells (Stuart‐Tilley et al., 1994; Xue et al., 2011). During migration, basolateral membrane proteins, including NHE1, translocate to the leading edge, while apical proteins may stay diffusely in the membrane. Migrating fibroblasts, immune cells, and tumor cells display high NHE1 activity in the lamellipodium, and directional movement of these cells critically depends both on NHE1 transport function and on its molecular interaction with actin filaments via ERM domain proteins (Denker & Barber, 2002; Stock et al., 2005). NHE1 is critically involved in modulating initial steps in integrin signalling for the assembly of focal adhesions (Tominaga & Barber, 1998), and more recent studies suggest the formation of pH nanodomains through NHE1 mediated proton export at the focal adhesion contacts in human melanoma cells (Ludwig, Schwab, & Stock, 2013). An extrusion of protons by NHE2 in another membrane area could reduce the proton concentration near the leading edge and the focal adhesions and reduce the activity of NHE1 and other acid/base transporters engaged in RGM1 cell migration such as MCTs and NBCs. Ragasa et al. (2007) suggested that the inhibitory effect of the stilbene DIDS on RGM1 cell migration may be due to its inhibitory potency for monocarboxylate transporters (MCTs). We recapitulated these experiments (suppl. Fig. 8A–C) and found the same strong inhibition of restitution by DIDS. Gallagher, Castorino, and Philp, (2009) found that the basolaterally located MCT4 and, to some extent, MCT1 to interact with beta integrins at the focal adhesions during cell migration. It is therefore, conceivable, but has not been tested, that MCTs rather than, or in addition to, NHE1 mediate the formation of pH microdomains at the focal adhesions in RGM1 cells. It is therefore, feasible that NHE2‐mediated proton extrusion away from the leading edge is a physiological mechanism to curb migratory speed and facilitate epithelial re‐differentiation after the wound has been closed.

Utilizing NHE2 knockout mice and WT littermates, Xue et al. (2011) demonstrated both an apical location and an involvement of NHE2 in trefoil factor‐stimulated epithelial wound healing. Other studies have investigated the impact of NHE2 on restitution in ischemia‐induced injury of the small intestine: NHE2 was suggested to be involved in tight junction formation, which would fit with our results suggesting that slowing of the migration process may be necessary to establish optimal barrier function (Moeser, Nighot, Ryan, Wooten, & Blikslager, 2006; Moeser et al., 2008). In Caco‐2 cells, stress‐induced barrier dysfunction was prevented by NHE2 inhibition (Nowak et al., 2004). All of the above mentioned studies utilized either pharmacological inhibition of NHE2 (which will always include NHE1 inhibition) or utilized NHE2 KO mice, which have alterations in the epithelial structure (Schultheis et al., 1998). We therefore, believe that the present study adds an important molecular detail to the understanding of the effect of NHE2 in gastrointestinal physiology. Clearly, not enough factors that regulate migratory behaviour in epithelial cells on a molecular basis during “sheet migration” are understood. It will be an important task for the future to construct and study heterologous expression systems for NHE2, similar to those that allowed extensive characterization of NHE3 function and regulation (reviewed in (Alexander & Grinstein, 2009; Donowitz et al., 2009; He & Yun, 2010), before we will fully understand the role and regulation of this NHE isoform in the gastrointestinal mucosa.

## Supporting information

Additional Supporting Information may be found online in the supporting information tab for this article.

Supporting Data S1.
