# Supplementary material for: Na+/H+ exchanger NHE1 and NHE2 have opposite effects on migration velocity in rat gastric surface cells
Source: J Cell Physiol. 2017 Feb 21;232(7):1669–80. doi: 10.1002/jcp.25758 (PMC5396337; doi:10.1002/jcp.25758)
Supplement: Supplementary file 1 — Supporting Data S1. [file JCP-232-1669-s001.pdf]

Supplementary Table 1.

	sequence (5‘ → 3‘)	concentration	efficiency
**NHE1**	for: ACCACTGGGCCTTTCTGGGGTT<br>rev: AAGGGTGGAGCTCTGACTGGCA	300nM	2.044
**NHE2**	for: CGCGAGCGCAGGGCTTCTAC<br>rev: CCTGGCCCTGGGCTGCAAAT	40nM	1.974
**NHE2tv1**	for: GGGACAACGCCGTTCGAGGAAA<br>rev: ACAGCTCTCCGGCACAATCGT	50nM	1.973
**bActin**	for: CAACCTTCTTGCAGCTCCTCCGT<br>rev: CCATACCCACCATCACACCCTGGT	300nM	1.915
**RPS9**	for: AGTCAACATGCCGGTCGCCAGA<br>rev: AGTCGTCTCAGCAGAGCGTTGC	500nM	2.031

Supplementary Table 1: Information on primers for quantitative PCR.

The table displays primer sequence, concentration, and efficiency for rat primers in RGM1 cells. There are two known transcript variants of rat NHE2: the NHE2tv1 primer pair detects the longer transcript variant 1. The NHE2 primer pair binds to both transcript variants.

Supplementary Table 2.

	1x NaCl solution		1x NH_4Cl solution		1x TMACl solution		1x high K^+ clamp solution	
**In the presence of**	O_2	CO_2	O_2	CO_2	O_2	CO_2	O_2	O_2*
**Na^+ [mEq]**	130	120	-	80	-	-	-	22
**Cl^- [mEq]**	141	96	141	96	141	96	141	40
**K^+ [mEq]**	6	5.25	6	5.25	6	5.25	130	123.25
**Mg^{2+} [mEq]**	1	1.2	1	1.2	1	1.2	1	1.2
**Ca^{2+} [mEq]**	2	1.2	2	1.2	2	1.2	2	1.2
**NH_4^+ [mEq]**	-	-	32	20	-	-	-	-
**HPO_4^{2-} [mEq]**	0.5	3.75	0.5	3.75	0.5	3.75	0.5	3.75
**SO_4^{2-}**	-	1.2	-	1.2	-	1.2	-	1.2
**HCO_3^-**	-	24	-	24	-	24	-	-
**gluconate$^-$ [mEq]**	-	2.4	-	2.4	-	2.4	-	102.4
**choline$^+$ [mEq]**	-	-	-	-	-	-	-	24
**TMA^+ [mEq]**	-	-	98	20	130	96	-	-
**HEPES [mM]**	11	10	11	10	11	10	11	10
**TRIS [mM]**	-	5	-	5	-	5	-	-
**D-glucose [mM]**	25	10	25	10	25	10	25	-

Supplementary Table 2: Information on solutions used to measure intracellular pH.

This table presents the composition of solutions that were used to measure the intracellular pH of RGM1 cells. 1x NaCl solution was used during BCECF staining, determination of steady-state pH, as well as prior to and after intracellular acid load. The intracellular acid

load was achieved using 1x NH4Cl and 1x TMACl solution. 1x high potassium clamp solution was applied at two known pH values to convert BCECF emission signal to pH values by linear curve fitting. Solutions were gassed with either 100% O_2 ("O_2") or 5% CO_2 and 95% O_2 ("CO_2") as indicated in the last line of the table. O_2*: This high potassium clamp solution was gassed with 100% O_2 and it was used for all experiments in which the prior solutions were gassed with 5% CO_2 and 95% O_2.

Supplementary Figure 1.

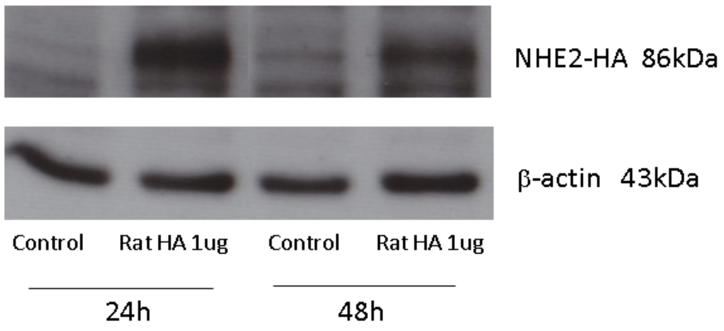

Supplementary Figure 1: Western Analysis of the lentivirally expressed NHE2-HA gene product.

HEK293 cells were recovered 24 hours, and 48 hours after transfection, lysed, and Western analysis was performed using an anti HA antibody. A band of the predicted size for HA NHE2 (~91.5 kD) was detected only in the NHE2-HA vector transfected cells.

Supplementary Figure 2.

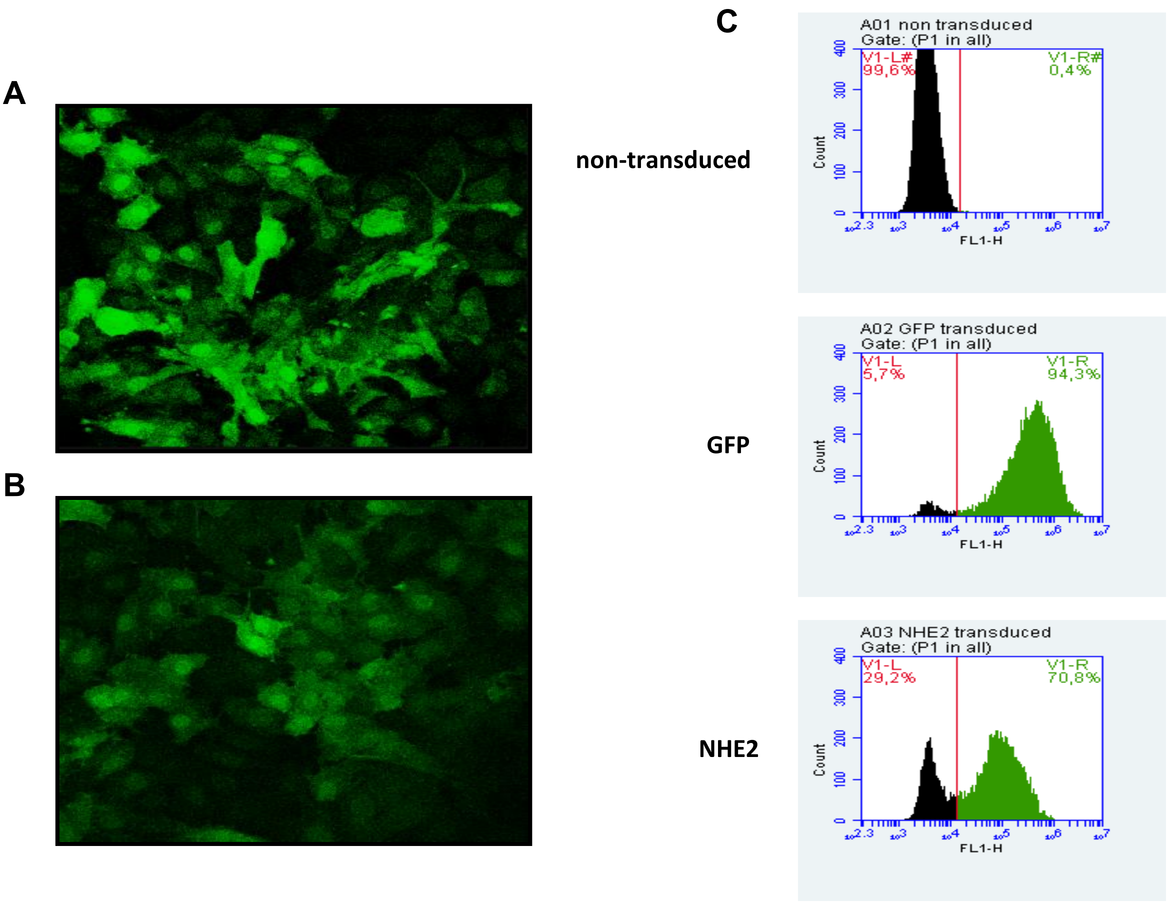

Supplementary Figure 2: Transient lentiviral overexpression of rat NHE2 in RGM1 cells.

HA-tagged rat NHE2 (HA-rNHE2) was overexpressed in RGM1 cells by transient lentiviral transduction. A, B: To assure transduction with similar amounts of control virus and HA-rNHE2 lentivirus, similar GFP detection was sought in confocal microscopy at 488nm excitation wavelength. Cells were transduced with 200µl non-concentrated control lentivirus per $0.1x10^6$ cells (A) and 20µl concentrated HA-rNHE2 containing virus (B). C: Flow cytometry shows that a good portion of RGM1 cells was successfully transduced by both the GFP containing control vector (GFP) as well as the HA rNHE2 containing vector (NHE2).

Supplementary Figure 3.

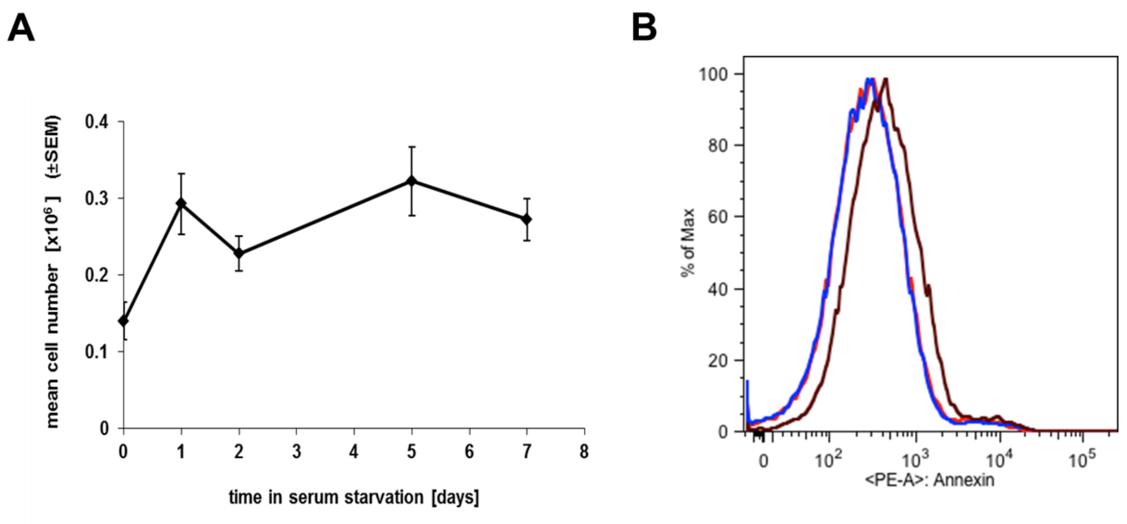

Supplementary Figure 3: Serum-free medium arrests RGM1 proliferation without inducing apoptosis.

A: To investigate whether RGM1 proliferation was arrested by withdrawing growth factors, RGM1 cells were grown in 0% FCS medium for several days, and medium changes were performed at least every second day. Trypan blue staining was used to exclude dead cells during subsequent counting. Cells were totaled on a hemocytometer at the beginning of the experiment (day 0) and after 1, 2, 5, and 7 days. Proliferation was eliminated after 24 hours in 0% FCS medium. Data are presented with SEM.

B: Apoptosis was analyzed by PE Annexin V Apoptosis Detection Kit (BD Biosciences) according to the instructions. Briefly, RGM1 cells grown for 24 h under the different conditions, were stained with Annexin V to detect cells with surface phosphatidylserine expression, an indicator for apoptosis or cell death, as well as 7-AAD, a cell membrane impermeable dye which will only stain late apoptotic and dead cells (results not shown). As expected for adhesive epithelial cells, there were some dead cells after trypsinization in all of the experiments (peak absorbance), but no difference in apoptotic cells between RGM1 cells grown in 20% FCS (brown), 1% FCS (blue) or 0% FCS (red).

Supplementary Figure 4.

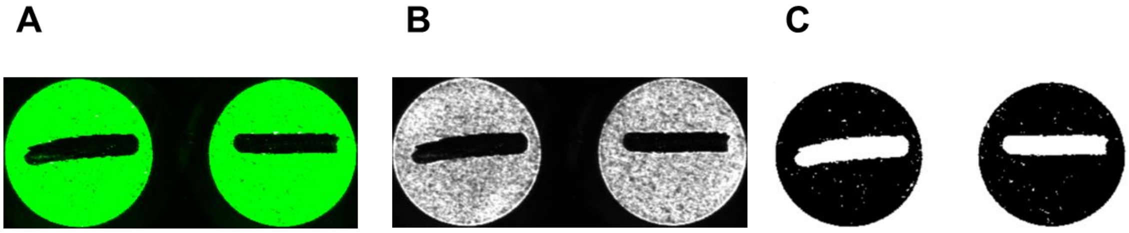

Supplementary Figure 4: Thresholds in initial scans were adjusted to properly depict outlines of the scratches while not picking up unspecific reflections.

This figure shows the scan of two wells of a 96 well plate one hour after scratching (initial scan). A: The first part shows a false color image of the fluorescent scan acquired by the Odyssey 2.1 program. The false color image makes it easy to distinguish the “green” cells from the “black” scratch. B: The second part displays the same scan converted to an 8-bit file with the ImageJ program. C: In the third part of this figure, a threshold was adjusted for the 8-bit greyscale file to convert all grey values below the threshold to white and all above the threshold to black. On this basis a black and white image was created and the area covered by white pixels, representing the scratch, was measured automatically by ImageJ.

While the right well of A, B, and C demonstrates a nice staining without unspecific reflections within the scratch, the left well illustrates some minor reflections within the “wound”. However, after adjusting the threshold, the outlines of the wounds are depicted properly without picking up those minor reflections (C).

Supplementary Figure 5.

A

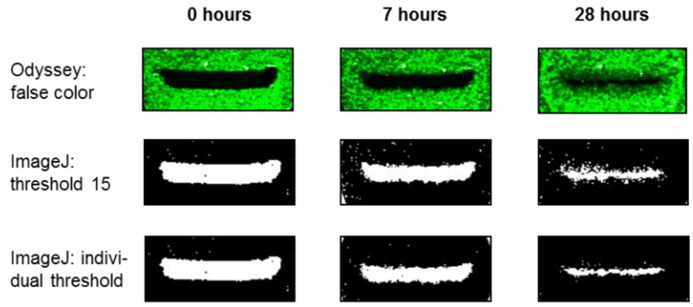

B

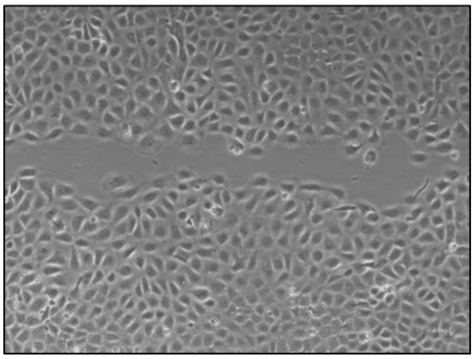

Supplementary Figure 5: An individual threshold had to be adjusted for each well at later time points to still properly depict the outlines of the wounds**.**

A: This figure focuses on an exemplary scratch that is displayed in false color with the Odyssey program. Scans were taken one hour after scratching, i.e. at the beginning of the migration assay (0 hours), and after another seven and 28 hours.

Cell spreading during migration locally reduced the monolayer density, which in turn led to weaker emission signals at the edges of the wound. This weaker signal can best be observed in the Odyssey false color image after 28 hours, but is also already visible after seven hours: the emission signal at the wound margins turned from bright green (at the beginning of the assays) to dark green. These migrations were performed in medium containing 0% FCS. In 20% FCS the wound healing velocity will be even faster, thus further reducing the emission signal at the edges of the wounds. Hence, an ImageJ threshold that properly displays the bright edges in the initial scan will not accurately outline the darker, migrating margins at later points in time (ImageJ: threshold 15). Consecutively, individual ImageJ thresholds were adapted for each well at later points in time to account for the reduced light emission (ImageJ: individual threshold). Here, thresholds 15, 12, and 9 were chosen for 0, 7, and 28 hours migration time.

B: This image was taken at 100x magnification (working group Schwab, Physiology, University Muenster). RGM1 cells were grown to a confluent monolayer in a T12.5 flask and scratched with a 100µl tip. After a medium change and a one hour recovery at 37°C and 5% CO_2, the air-tight lid of the flask was closed to keep the 5% CO_2 atmosphere. Then, the flask was placed into a heated chamber installed on an inverted microscope. This image was taken after three hours of migration. Inspecting RGM1 migration shows several rows of polarized cells at the front of the migrating sheet, whereas the monolayer far back remains mostly compact. This migrating sheet explains the reduced light emission at the scratch margins in the fluorescence-based scratch assay.

Supplementary Figure 6.

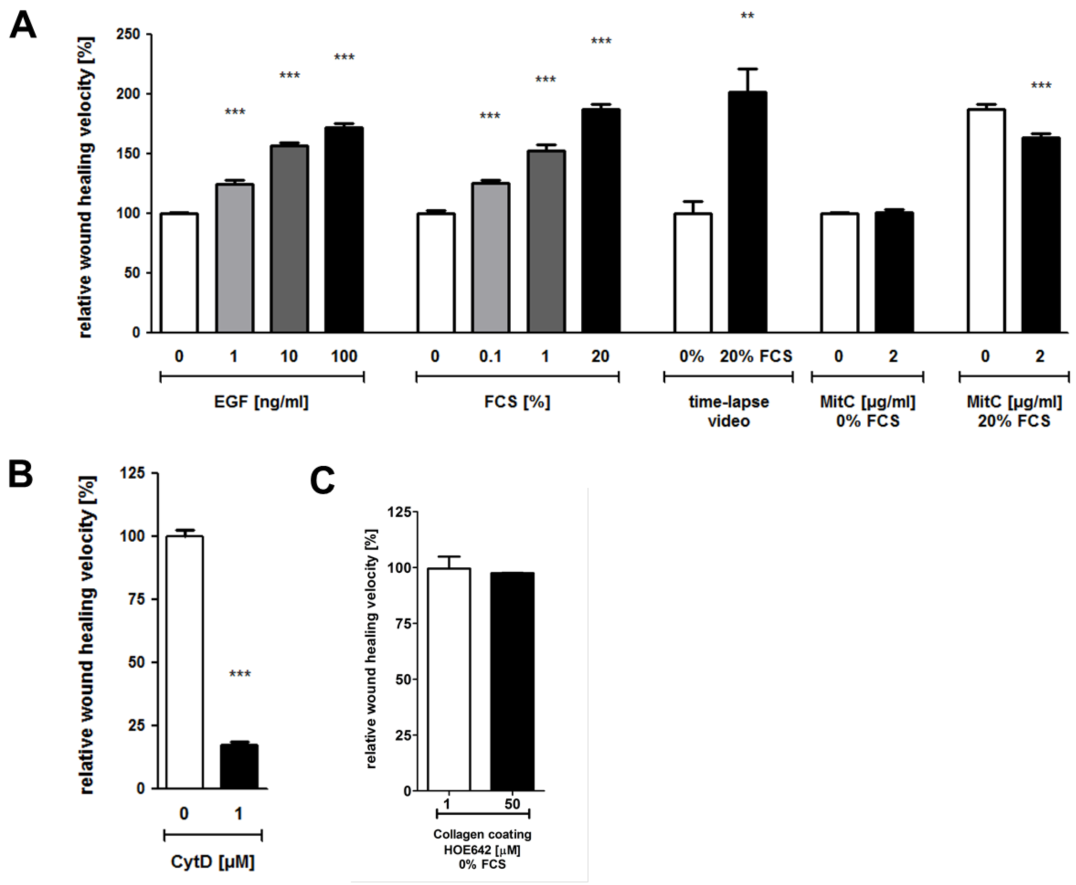

Supplementary Figure 6: The fluorescence-based scratch assay can properly depict induction and inhibition of RGM1 wound healing.

RGM1 wound healing velocity was increased or decreased by known inducers or inhibitors to validate the new fluorescence-based wound healing assay. Bars show the relative wound healing velocity in percent compared to the respective control with 0% FCS (white bars). Data are presented with SEM. A: Wound healing was induced dose-dependently by epidermal growth factor (EGF) and fetal calf serum (FCS) in RGM1 cells. Additionally, RGM1 were employed in a well-established scratch assay, based on time-lapse video microscopy, where FCS sped up wound closure to the same extent as in our fluorescence-based assay (time-lapse video). In an additional set of experiments, cytostatic DNA crosslinker Mitomycin C (MitC) was applied to inhibit cell proliferation. RGM1 cells were preincubated for two hours with 2µg/ml MitC. MitC did not change wound healing velocity in 0% FCS, supporting the idea that RGM1 cells cannot proliferate in the absence of serum, whereas wound closure was reduced by MitC when cells were kept with 20% FCS. However, only a minor part of wound healing velocity is due to increased proliferation. The major part of wound closure is accounted for by cell migration. B: RGM1 wound healing was inhibited by Cytochalasin D (CytD), an inhibitor of actin assembly and inducer of actin depolymerisation. Statistical significance was determined by comparison to the corresponding controls (white bars) using one-way ANOVA with Dunnett's post test and two-tailed t-test for time-lapse videos and CytD, respectively. ** $p < 0.01$, *** $p < 0.001$.

Supplementary Figure 7.

A B

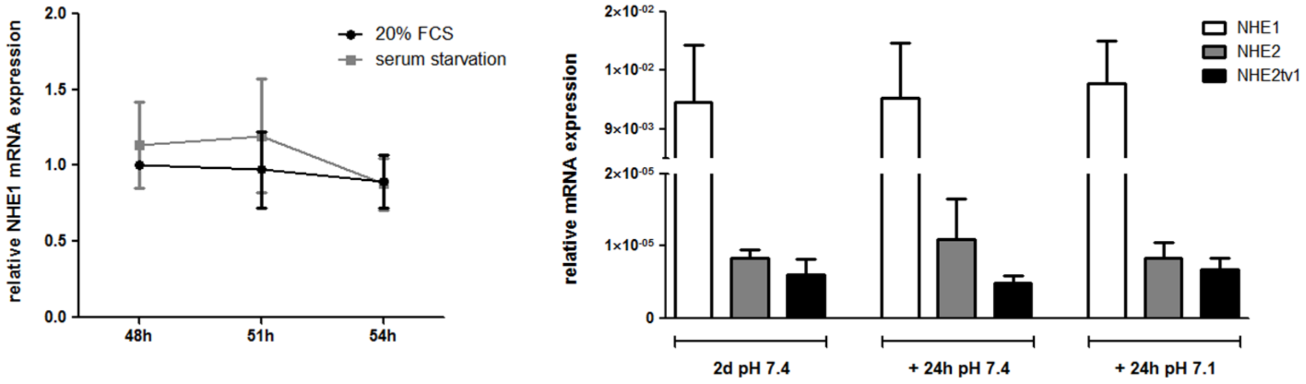

Supplementary Figure 7: FCS concentration of the cell culture medium and distinct changes in extracellular pH does not affect NHE1 mRNA expression levels.

A: Graph shows the NHE1 mRNA expression levels of RGM1 cells relative to the reference gene index (geometric mean of efficiency corrected Cq values of RPS9 and bActin). RGM1 cells were grown in medium supplemented with 20% FCS. NHE1 mRNA expression was determined after two days, mimicking the beginning of the wound healing assay (20% FCS, 48h), or another 3 and 6 hours later, mimicking the middle and the end of the wound healing assay (20% FCS, 51h and 54h). In the second experiment, RGM1 cells were deprived of serum prior to mRNA measurements (serum starvation, 48h), and afterwards 20% FCS was re-added for 3h and 6h (serum starvation, 51h and 54h). However, no significantly different NHE1 expression was seen in the presence, absence, and after re-addition of FCS. Data are presented with SEM.

B: Graph shows the NHE mRNA expression levels of RGM1 cells relative to the reference gene index (geometric mean of efficiency corrected Cq values of RPS9 and b-Actin). RGM1 cells were grown in medium supplemented with 20% FCS for two days (2d pH 7.4), afterwards they were additionally incubated for 24 hours at pH 7.4 (+24h pH 7.4) or pH 7.1 (+24h pH 7.1). No significant change in NHE1 expression was observed. NHE2 expression was hardly detectable in the RGM1 cell line, neither with nor without acidic pH. The NHE2tv1 primer pair detects only full length rat NHE2, the NHE2 primer pair detects both NHE2 transcript variants. Data are presented with SEM. Statistical significance was determined by one-way ANOVA with Bonferroni‘s multiple comparison post test.

Supplementary Figure 8.

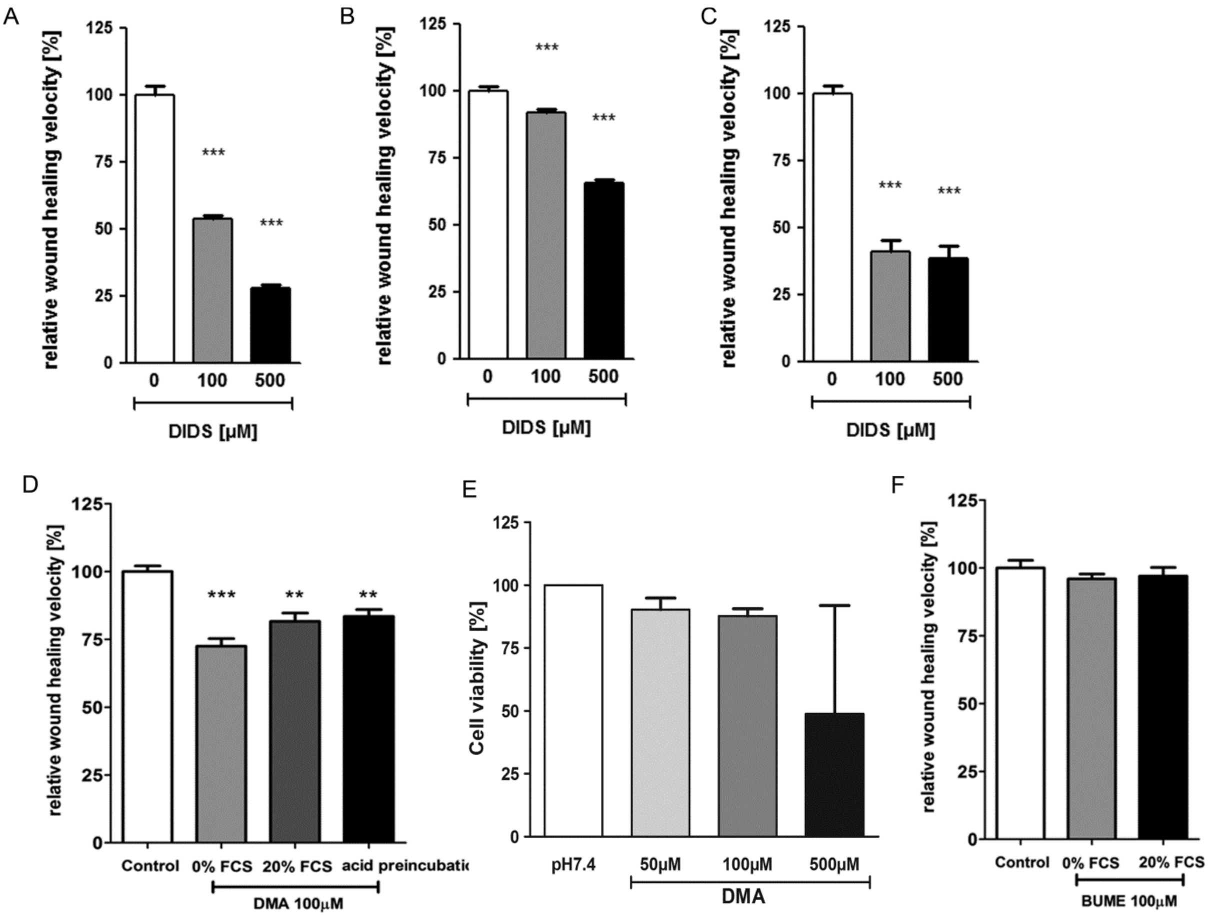

Supplementary Figure 6: The stilbene DIDS and the amiloride analogue DMA inhibit RGM1 wound healing.

RGM1 wound healing was performed (A) in the absence of growth factors during the assay (0% FCS), (B) in the presence of growth factors (20% FCS), and (C) after 24 hour incubation with pH 7.1 prior to the assay, in the absence and presence of 100µM and 500µM of the stilbene DIDS. DIDS had a stronger inhibitory effect on nonstimulated or acid-stimulated wound healing than on FCS-stimulated wound healing, suggesting that the effect may be in part due to non-transporter related effects, as suggested by Ragasa et al. (2007). D: Wound healing velocity was determined in the absence and presence of 100µM dimethyl amiloride (DMA), both in 0 and 20% FCS and after acid preincubation. Each bar is the percent of wound healing velocity in percent of its own vehicle control (100%). E: WST cell viability after incubation with DMA. F Wound healing velocity in the presence and absence of 100µM bumetanide (no effect). As seen with DIDS, the strongest effect of DMA was seen under nonstimulated conditions, where NHE1 is likely quiescent and HOE642 had no effect. DMA is known to have a variety of effects besides Na/H exchange inhibition (Kleymann & Cragoe, 1988, Frelin et al. 1988) , which may explain the early results showing a strong dependence of restitution on Na/H exchange.Statistical significance was determined by comparison to the corresponding controls (white bars) using one way ANOVA with Dunnett's post test. *** $p < 0.001$

Supplementary Figure 9.

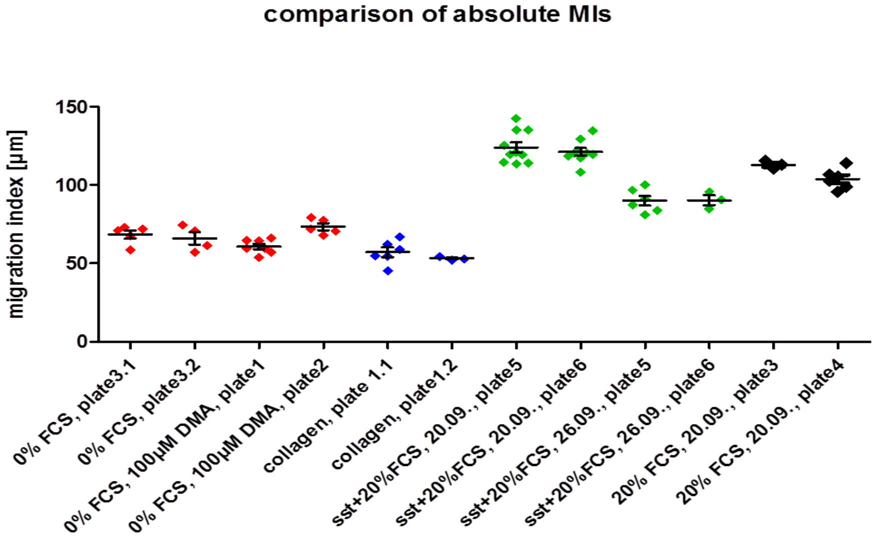

Supplementary Figure 9: To determine wound healing velocity, one has to know the area covered by migrating cells first. This so-called Δarea was calculated as: Δarea = initial wound area – final wound area. However, it is not possible to create wounds of the same size each time. Therefore, in bigger scratches, more cells will move at the same time, resulting in larger Δareas for bigger wounds. There is a strong positive correlation between the initial area of the wound and Δarea, but there is an even stronger correlation between the initial perimeter and Δarea. To be independent of the initial wound size, wound healing velocity was analyzed as migration index: MI = Δarea / initial perimeter. The above figures shows absolute migration indicates in an initial series of test experiments. It is evident that reproducibility withIn the same experimental set is excellent, but there is sometimes variation from one cell passage to the next. In the manuscript, the data were expressed in relative values with controls converted to 100% to account for possible inter-assay variation.
